# Pharmacogenetic Associations Between Atazanavir/UGT1A1*28 and Efavirenz/rs3745274 (*CYP2B6*) Account for Specific Adverse Reactions in Chilean Patients Undergoing Antiretroviral Therapy

**DOI:** 10.3389/fphar.2021.660965

**Published:** 2021-05-19

**Authors:** Daniela Poblete, Fernando Bernal, Gabriel Llull, Sebastian Archiles, Patricia Vasquez, Leonardo Chanqueo, Nicole Soto, María A. Lavanderos, Luis A. Quiñones, Nelson M. Varela

**Affiliations:** ^1^ Laboratory of Chemical Carcinogenesis and Pharmacogenetics, Department of Basic and Clinical Oncology, Faculty of Medicine, University of Chile, Santiago, Chile; ^2^ Department of Infectious Diseases, Hospital San Juan de Dios, Santiago, Chile; ^3^ Clinical Laboratory, Hospital San Juan de Dios, Santiago, Chile; ^4^ Latin American Network for the Implementation and Validation of Clinical Pharmacogenomics Guidelines (RELIVAF-CYTED), Madrid, Spain

**Keywords:** UGT1A1, CYP2B6, ADRs, atazanavir, efavirenz, HIV, pharmacogenetic, antiretroviral

## Abstract

**Background:** Efavirenz (EFV), a non-nucleoside reverse transcriptase inhibitor, and atazanavir (ATV), a protease inhibitor, are drugs widely used in antiretroviral therapy (ART) for people living with HIV. These drugs have shown high interindividual variability in adverse drug reactions (ADRs). UGT1A1*28 and *CYP2B6* c.516G>T have been proposed to be related with higher toxicity by ATV and EFV, respectively.

**Objective:** To study the association between genetic polymorphisms and ADRs related to EFV or ATV in patients living with HIV treated at a public hospital in Chile.

**Methods:** Epidemiologic, case–control, retrospective, observational study in 67 adult patients under EFV or ATV treatment was conducted, in the San Juan de Dios Hospital. Data were obtained from patients’ medical records. Genotype analyses were performed using rtPCR for rs887829 (indirect identification of UGT1A1*28 allele) and rs3745274 (*CYP2B6* c.516G>T), with TaqMan® probes. The association analyses were performed with univariate logistic regression between genetic variants using three inheritance models (codominant, recessive, and dominant).

**Results:** In ATV-treated patients, hyperbilirubinemia (total bilirubin >1.2 mg/dl) had the main incidence (61.11%), and moderate and severe hyperbilirubinemia (total bilirubin >1.9 mg/dl) were statistically associated with UGT1A1*28 in recessive and codominant inheritance models (OR = 16.33, *p* = 0.028 and OR = 10.82, *p* = 0.036, respectively). On the other hand, in EFV-treated patients adverse reactions associated with CNS toxicity reached 34.21%. In this respect, nightmares showed significant association with *CYP2B6* c.516G>T, in codominant and recessive inheritance models (OR = 12.00, *p* = 0.031 and OR = 7.14, *p* = 0.042, respectively). Grouped CNS ADRs (nightmares, insomnia, anxiety, and suicide attempt) also showed a statistically significant association with *CYP2B6* c.516G > T in the codominant and recessive models (OR = 30.00, *p* = 0.011 and OR = 14.99, *p* = 0.021, respectively).

**Conclusion:** Our findings suggest that after treatment with ATV or EFV, UGT1A1*28 and *CYP2B6* c.516G>T influence the appearance of moderate-to-severe hyperbilirubinemia and CNS toxicity, respectively. However, larger prospective studies will be necessary to validate these associations in our population. Without a doubt, improving adherence in patients living with HIV is a critical issue to the success of therapy. Hence, validating and applying international pharmacogenetic recommendations in Latin American countries would improve the precision of ART: a fundamental aspect to achieve the 95–95–95 treatment target proposed by UNAIDS.

## Introduction

Human immunodeficiency virus (HIV) affects up to 38 million people worldwide, and it is considered a pandemic. In 2010, HIV/acquired immunodeficiency syndrome (AIDS) was the eighth cause of death, but in 2019, it was the 19th. It means that prevention, detection, and treatment initiatives have been successful in recent decades. But in recent years, the World Health Organization (WHO) reported a general slowdown in progress against infectious diseases such as HIV, tuberculosis, and malaria. In that context, the Joint United Nations Programme on HIV/AIDS (UNAIDS) proposed an ambitious goal: the 95–95–95 treatment target. It means that in 2030, 95% of all people living with HIV will know their HIV status, 95% of all people with diagnosed HIV infection will receive sustained antiretroviral therapy, and 95% of all people receiving antiretroviral therapy will have viral suppression (ONUSIDA, 2014). Currently, in Chile, according UNAIDS, we have reached 90-68-62 (ONUSIDA, 2019).

Antiretroviral therapy (ART) with associations of three drugs has a great impact. It is highly effective in suppressing viral replication (Moore et al., 2005), allows the qualitative and quantitative recovery of the immune response (Gras et al., 2007), prevents clinical progression, and significantly reduces associated mortality, despite there being no eradication of the virus (FrankPalella et al., 2003). The generally accepted formula for a potent antiretroviral regimen includes two nucleoside reverse transcriptase inhibitors (NRTIs) added to a third from another family: a non-nucleoside reverse transcriptase inhibitor such as efavirenz (EFV), a protease inhibitor such as atazanavir (ATV), or integrase inhibitor such as raltegravir (MINSAL, 2013). Recently, in Chile, there has been an increase in the prescription of integrase inhibitors, thus reducing the prescription of EFV and ATV. This is mainly due to the safety criteria, since it is expected that patients will have fewer adverse drug reactions (ADRs) associated with ART.

To improve the current HIV situation in our country, therapeutic adherence to ART becomes relevant, considering that ADRs are one of the main causes of treatment abandonment. For example, 5–10% of patients receiving ATV discontinue treatment due to ADRs, whereas at least 50% of patients receiving EFV present ADRs (Gaida et al., 2016). This variability is based on the characteristics of the drug and the patient. At the last point, the patient's own genetics helps to explain the interindividual variability in the presence of adverse events (Roden and George, 2002; Quiñones et al., 2017).

The Clinical Pharmacogenetics Implementation Consortium (CPIC; https://cpicpgx.org/) is an international consortium of individual volunteers and staff who are interested in facilitating the use of pharmacogenetic test for patient care. Its goal is to address this barrier to clinical implementation of pharmacogenetic test by creating, curating, and posting freely available, peer-reviewed, evidence-based, updatable, and detailed gene/drug clinical practice guidelines (https://cpicpgx.org/guidelines/). Each clinical guide provides information to allow the interpretation of clinical genotype test so that the results can be used to inform the prescribing of drugs (Caudle et al., 2014).

EFV and ATV, drugs widely used in ART in people living with HIV, have shown to possess high interindividual variability in ADRs. EFV is predominantly metabolized by CYP2B6, a highly polymorphic enzyme. Patients with certain genetic variants may be at increased risk of developing ADRs at the central nervous system (CNS) level. The single-nucleotide polymorphism (SNP) *CYP2B6* c.516G>T (NM_000767.5; NG_007929.1:g.20638G > T; rs3745274; *p*. Q172H) has been extensively studied. All alleles carrying c.516G>T [e.g., CYP2B6*6, in haplotype with c. A785G (Manosuthi et al., 2014)] are considered to lead to a reduced enzyme activity (Hofmann, et al., 2008). There is substantial evidence that links this SNP (rs3745274) with increase in plasma EFV concentrations (Sukasem et al., 2013; Desta et al., 2019). In this case, for these patients, there is a moderate recommendation from the CPIC guideline to consider starting efavirenz with a reduced dose of 400 or 200 mg/day. This recommendation reflects the fact that most CYP2B6 poor metabolizers do not discontinue efavirenz 600 mg/day due to ADRs (Desta et al., 2019).

On the other hand, ATV inhibits hepatic uridine diphosphate glucuronosyltransferase (UGT)1A1, the major UGT1A subfamily enzyme, expressed primarily in the liver and gastrointestinal tract (Court et al., 2012), and is essential for the metabolization (conjugation) of bilirubin and some drugs (Bosma et al., 1994; Hongkaew et al., 2018). Reduced UGT1A1 activity either through genetic variation (Kadakol et al., 2000; Strassburg, 2008) or catalytic inhibition by drugs (Zhang et al., 2005) results in the accumulation of unconjugated (indirect) bilirubin in blood and tissues. Mechanistic studies using promoter–reporter constructs have shown that the presence of a TATAA consensus element in *UGT1A1* promotor containing seven TA dinucleotide repeats (TA_7_ or UGT1A1*28 allele) causes reduction in gene transcription as compared with the reference TA_6_ (UGT1A1*1) allele (Bosma et al., 1995), possibly due to reduced binding affinity for transcription factors including TATA-binding protein (Hsieh et al., 2007). Thereby, the presence of ATV prevents the glucuronidation and elimination of bilirubin; resultant indirect hyperbilirubinemia can cause jaundice and a potential discontinuation of atazanavir treatment. Risk for bilirubin-related discontinuation is highest among individuals who carry UGT1A1*28, decreased function alleles. The CPIC guideline suggests a *UGT1A1* genotype is most helpful if available before atazanavir is prescribed (Gammal et al., 2016). Due to the methodological complexity involved in determining the presence of UGT1A1*28, the majority of population studies apply an indirect identification strategy, identifying a SNP located less than 300 base pairs from the TA repeat, rs887829 (c. −364C>T; UGT1A1*80), where the T allele is in a very strong linkage disequilibrium (LD) with the TA_7_ allele (UGT1A1*28) (*r* ^2^ ≅ 0.99) (Gammal et al., 2016). It is important to highlight that UGT1A1*6 is also associated to a reduced enzymatic function and could be relevant (Sukasem et al., 2016); however, the frequency in populations of non-Asian descent is extremely low (1,000 Genome) to be included in this study.

Since UGT1A1*28 (rs8175347, TA_7_) and *CYP2B6 c.516G>T* (rs3745274) have been proposed to be related with higher toxicity by ATV and EFV, respectively, the main goal of this research was to study the association between EFV or ATV ADRs and the presence of these genetic polymorphisms in patients from a public hospital in Chile.

## Patients and Methods

### Patients

We carried out an epidemiologic, case–control, retrospective, observational study in eighty patients. The inclusion criteria were applicable for the following patients: 1) ≥18 years old; 2) under EFV (600 mg/day) or ATV (300 mg/day with 100 mg/day of ritonavir as a booster) treatment; 3) with A1, A2, B1, or B2 (patients without AIDS) clinical category (outpatients) according to the Chilean HIV Clinical Guide (MINSAL, 2013) in “San Juan de Dios Hospital” (SJDH) (Santiago, Chile); 4) adherent to treatment; and 5) with the clinical record available for reviewing. Exclusion criteria were the following: 1) patients with pre-ART diagnoses of kidney or liver insufficiency, 2) patients under voriconazole treatment, 3) existence of untreated active opportunistic disease susceptible to severe Immune Reconstitution Inflammatory Syndrome (IRIS) with the initiation of ART and/or *tuberculosis*, and 4) patients who do not respect the treatment and control protocol established by the hospital unit. Data were obtained from patients’ medical records and organized in a coded database which included patient identification, clinical record number, sex, age (years), weight (kilograms), lymphocyte count (cells/mm^3^), viral loads (copies/mL), liver transaminases (U/L), total bilirubin level (mg/dl), serum creatinine levels (mg/dl), and lipid levels (total cholesterol, HDL, LDL, and triglycerides).

Before starting treatment with EFV, patients are evaluated by a psychologist to rule out preexisting CNS pathologies. Patients are a part of the Adherence Program of SJDH; it consists of a basal control before starting treatment, and then after two weeks, one month, and every three months. All controls include a session with a pharmacist and a physician, and allow the generation of records of ADRs to ART, which are classified according to their presence or absence, and results of clinical and hematological laboratory analyses. ADRs registered were hyperbilirubinemia (total bilirubin level >1.2 mg/dl), gastrointestinal upset, rash, dizziness, nightmares, insomnia, headache, fever, anxiety, lipodystrophy, dyslipidemia, and suicide attempt. Hyperbilirubinemia was classified for total bilirubin levels as follows: grade 1 (mild), 1.3–1.9 mg/dl; grade 2 (moderate), 1.9–3.1 mg/dl; grade 3 (severe), 3.1–6.1 mg/dl; and grade 4 (serious), >6.1 mg/dl (Fellay et al., 2001; MINSAL, 2013). Adherence to treatment was assessed by applying an adherence questionnaire (Knobel et al., 2002) and correlated with pharmacy withdrawals and viral load levels. The need for volunteer patients to agree to answer the questionnaire was established in the document associated with the informed consent procedure.

### Genotyping Analyses

Whole venous EDTA blood samples were obtained at the SJDH and analyzed in Laboratory of Chemical Carcinogenesis and Pharmacogenetics of the Faculty of Medicine, the University of Chile. The sample was immediately refrigerated at 4–6°C until centrifugation. Then the sample was centrifuged at 3,000 rpm at 4°C for 15 min. Subsequently, 250 μL of buffy coat was carefully removed from the interface and deposited in a properly labeled tube. The buffy coat sample was frozen at −80°C until DNA extraction with E.Z.N.A.® Blood DNA Mini Kit, OMEGA Bio-tek, Inc. (Norcross, Georgia, United States). Genomic DNA (gDNA) samples were quantified using a nanodrop Denovix® DS-11 FX Series Spectrophotometer (Wilmington, Delaware, United States). The absorbance ratio of 260/280 nm was used to evaluate the purity of the gDNA, considering a value higher than 1.7 as acceptable. The samples were genotyped using a Stratagene® Mx3000P Real-Time PCR system (Agilent Technologies, Waldbronn, Germany). The reaction mixture consisted of 30 ng of gDNA, 5.0 µL of *TaqMan*® Genotyping Master Mix 2X (Applied Biosystems™; catalog no. 4371355), 0.50 µL of *TaqMan*® SNP genotyping assay (Thermo Fischer Scientific Inc.®), and nuclease-free water (sufficient quantity for 10 uL). The thermal cycling conditions were as follows: an initial denaturation and enzyme activation at 95°C for 10 min, followed by 50 cycles, each consisting of two phases: denaturation at 95°C for 15 s and annealing/extension at 60°C for 90 s. For the determination of *CYP2B6* c.516G>T (rs3745274), the *TaqMan*® SNP genotyping assay catalog no. 4362691 (assay ID: C_7,817,765_60) was used. For the indirect determination of the UGT1A1*28 allele, the identification of rs887829 is used as a strategy, through the *TaqMan*® SNP genotyping assay catalog no. 4351379 (assay ID: C_2,669,357_10).

### Statistical Analyses

The association analyses were performed with univariate logistic regression between genetic variants using three inheritance models (codominant, recessive, and dominant) on STATA 12.0 (Copyright 1985–2011 StataCorp, LP, TX, United States), and a *p-value* < 0.05 was considered statistically significant. All association studies were assayed by testing three genetic models of inheritance, that is, dominant, codominant, and recessive models, and choosing parameters with better statistical association for each analysis. χ^2^ and Fisher`s exact test were applied to compare proportions between independent groups.

### Ethical Issues

The study was carried out under strict ethical procedures approved by the Ethics Committee of the Faculty of Medicine of the University of Chile (September 5, 2016) and authorized by the director of the SJDH (September 23, 2016) resolution No. 4855, in accordance with the procedures suggested in the Declaration of Helsinki (Declaration of Helsinki, 1964), and according to Chilean Laws 20,120, 20,584, and 19,628, and the guidelines of the Good Clinical Practices (GCP). All patients signed informed consent.

## Results

Table 1 shows baseline characteristics of the 67 Chilean patients who live with HIV. The mean age was 35.7 ± 10.4 years, median age was 34 years, and the age group from 30 to 39 years (35.82%) was the most prevalent. 54 patients were males (80.6%) and 13 females (19.4%). Our patients were treated with different pharmacological schemes from their diagnosis; 31 patients were treated with EFV, 29 used ATV, and 7 patients first received EFV or ATV in a specific scheme and afterward changed to ATV or EFV, respectively, or *vice versa*, so they were included individually on ATV and EFV statistical analysis. Antiretroviral drugs (NRTIs) that were administered concurrently with EFV included abacavir/lamivudine (*n* = 21; 55.26%), tenofovir/emtricitabine (*n* = 13; 34.21%), and zidovudine/lamivudine (*n* = 4; 10.53%); and the ATV-treated patients completed their tritherapy with abacavir/lamivudine (*n* = 24; 66.67%), tenofovir/emtricitabine (*n* = 10; 27.78%), and zidovudine/lamivudine (*n* = 2; 5.56%).

**TABLE 1:** Baseline characteristics of the patients (*n* = 67).

Age	Years
Mean ± *SD*	35.7 ± 10.4
Median	34
**Age-group (years)**	**n (%)**
20–29	23 (34.33)
30–39	24 (35.82)
40–49	15 (22.39)
≥50	5 (7.46)
**Sex**	**n (%)**
Female	13 (19.40)
Male	54 (80.60)
**ART**	**n (%)^a^**
Patients treated with ATV	36
+ abacavir/lamivudine	24 (66.67)
+ tenofovir/emtricitabine	10 (27.78)
+ zidovudine/lamivudine	2 (5.56)
Patients treated with EFV	38
+ abacavir/lamivudine	21 (55.26)
+ tenofovir/emtricitabine	13 (34.21)
+ zidovudine/lamivudine	4 (10.53)

SD, standard deviation; n, number of patients; ART, antiretroviral therapy; EFV, efavirenz; ATV, atazanavir.

a Seven patients received EFV and ATV in different treatment regimens.

Twelve types of ADRs were registered from clinical records, and frequency was classified by the drug (Table 2). Hyperbilirubinemia had the main incidence (26.04% of all ADRs) and was observed in 61.11% of the patients treated with ATV. Gastrointestinal upset was observed in 18 patients (18.75%), and central nervous system (CNS) ADRs were present in 15 patients (15.63% of all ADRs).

**TABLE 2:** Type of adverse drugs reactions in patients undergoing antiretroviral therapy with EFV or ATV.

ADR	n	%	EFV-treated patients	ATV-treated patients
Hyperbilirubinemia^a^	25	26.04	3	22
Gastrointestinal upset	18	18.75	6	12
Rash	10	10.42	7	3
Dizziness	13	13.54	12	1
Nightmares	12	12.50	11	1
Insomnia	5	5.21	4	1
Headache	6	6.25	3	3
Fever	2	2.08	1	1
Anxiety	1	1.04	1	0
Lipodystrophy	1	1.04	1	0
Dyslipidemia	1	1.04	1	0
Suicide attempt	2	2.08	1	1
CNS^b^	15	15.63	13	2

ADR, adverse drugs reaction; CNS, central nervous system; EFV, efavirenz; ATV, atazanavir.

a Total bilirubin level >1.2 mg/dl.

b Nightmare, insomnia, anxiety, and suicide attempt were also grouped as CNS ADRs.

Although there were common adverse events in both groups of patients (treated with EFV or ATV), the presence of ADRs at the CNS level was significantly more frequent in the group of patients who received EFV (χ2: 9.3924; *p*: 0.002), while hyperbilirubinemia (total bilirubin level >1.2 mg/dl) was more frequent in patients who received atazanavir (χ2: 23.4030; *p*: <0.0001), considering that the distribution of antiretroviral drug regimens that accompanied ATV and EFV (Table 1) did not present significant differences between both groups (*p*: 0.592, Fisher’s exact test).

Table 3 shows genotypic and allelic frequency of polymorphisms in patients included in this study. Minor allele frequencies obtained for both polymorphisms were relatively high (*CYP2B6* c.516G>T, T: 0.38 and UGT1A1*28, TA_7_: 0.36), which allowed a good statistical potency for associations.

**TABLE 3:** Genotype and allele frequencies in patients recruited for this study.

Genetic variant	Genotypic frequency n (%)			Allelic frequency	
***CYP2B6* c.516G>T**	G/G	G/T	T/T	*f* G	*f* T
All patients (*n* = 67)	26 (38.81)	31 (46.27)	10 (14.92)	0.62	0.38
EFV-treated patients *(n* = 38)	14 (36.84)	18 (43.37)	6 (15.79)	0.61	0.39
UGT1A1*28 ^a^	TA_6_/TA_6_	TA_6_/TA_7_	TA_7_/TA_7_	*f* TA_6_	*f* TA_7_
All patients (*n* = 67)	31 (46.27)	24 (35.82)	12 (17.91)	0.64	0.36
ATV-treated patients (*n* = 36)	10 (27.78)	18 (50.00)	8 (22.22)	0.53	0.47

n, number of patients; f, frequency; EFV, efavirenz; ATV, atazanavir.

a Indirect identification of the UGT1A1*28 allele using rs887829.

Thirty-six patients were treated with ATV (Table 1); of the latter, only seven suffered severe hyperbilirubinemia (total bilirubin level >3.1 mg/dl). Univariate logistic regression analysis showed that the UGT1A1*28 allele was significantly associated with severe hyperbilirubinemia, in a recessive inheritance model (OR = 8.33, *p* = 0.023, 95% CI: 1.33–52.03). The other inheritance models could not be evaluated due to the absence of cases. None of the patients had serious hyperbilirubinemia (total bilirubin level >6.1 mg/dl).

Table 4 shows results for the univariate logistic regression analysis between the UGT1A1*28 allele and total bilirubin levels >1.9 mg/dl in patients treated with ATV; a statistically significant association was observed in the codominant and recessive inheritance models (OR = 16.33, *p* = 0.028 and OR = 10.82, *p* = 0.036, respectively). Headache, rash, and gastrointestinal upset did not show a statistically significant difference (data not shown).

**TABLE 4:** Univariate logistic regression between UGT1A1*28 (TA_7_) and total bilirubin level >1.9 mg/dl (moderate and severe hyperbilirubinemia) in patients treated with atazanavir.

Inheritance model	Genotypes^a^	OR	*p*-value	95% CI
Codominant model	TA_6_/TA_6_	1.00	Ref	-
	TA_6_/TA_7_	1.87	0.456	0.36–9.63
	TA_7_/TA_7_	16.33	0.028*	1.35–197.77
Recessive model	TA_6_/TA_6_ + TA_6_/TA_7_	1.00	Ref	-
	TA_7_/TA_7_	10.82	0.036*	1.17–100.44
Dominant model	TA_6_/TA_6_	1.00	Ref	-
	TA_6_/TA_7_ + TA_7_/TA_7_	3.18	0.146	0.67–15.15

OR: odds ratio; CI: confidence interval; ref: reference; *p-value < 0.05.

a Indirect identification of the UGT1A1*28 (TA_7_) allele using rs887829.

Same analysis was made on 38 patients treated with EFV. We did not find statistical significance among hyperbilirubinemia, gastrointestinal upset, rash, headache, insomnia, and dizziness, and polymorphisms included in this study (data not shown). However, nightmares had statistical significance on the codominant and recessive heritage model for *CYP2B6* c.516G>T (OR = 12.00, *p* = 0.031 and OR = 7.14, *p* = 0.042, respectively). No significance was observed in the dominant model (Table 5).

**TABLE 5:** Univariate logistic regression between *CYP2B6* c.516G>T (rs3745274) and nightmares in EFV-treated patients.

Inheritance model	Genotypes	OR	*p*-value	95% CI
Codominant model	G/G	1.00	Ref	-
	G/T	2.31	0.367	0.37–14.21
	T/T	12.00	0.031*	1.25–115.36
Recessive model	G/G + G/T	1.00	Ref	-
	T/T	7.14	0.042*	1.08–47.42
Dominant model	G/G	1.00	Ref	-
	G/T + T/T	3.6	0.142	0.65–19.90

OR, odds ratio; CI, confidence interval; ref, reference; *p-value < 0.05.

On the other hand, nightmare, insomnia, anxiety, and suicide attempt were grouped as CNS ADRs. The analysis of univariate logistic regression showed statistical significance on codominant and recessive heritage models with *CYP2B6* c.516G>T (OR = 30.00, *p* = 0.011 and OR = 14.99, *p* = 0.021, respectively). No significance was observed in the dominant model (Table 6).

**TABLE 6:** Univariate logistic regression between *CYP2B6* c.516G>T (rs3745274) and CNS toxicity (nightmares, insomnia, anxiety, and suicide attempt grouped) in EFV-treated patients.

Inheritance model	Genotypes	OR	*p*-value	95% CI
Codominant model	G/G	1.00	Ref	-
	G/T	3.00	0.229	0.50–17.95
	T/T	30.00	0.011*	2.19–410.99
Recessive model	G/G + G/T	1.00	Ref	-
	T/T	14.99	0.021*	1.52–148.31
Dominant model	G/G	1.00	Ref	-
	G/T + T/T	5.08	0.061	0.93–27.75

OR, odds ratio; CI, confidence interval; ref, reference; *p-value < 0.05.

## Discussion

We report here that the most frequently observed ADRs were hyperbilirubinemia, and gastrointestinal and CNS alterations (26.04, 18.75, and 15.63%, respectively). This pattern of ADRs is similar to that observed in a study carried out in 2011 in the same hospital; precisely, ADRs such as hyperbilirubinemia, and gastrointestinal and CNS alterations were the most frequent (19.7, 21.1, and 13.2%, respectively) (Bernal Ortiz et al., 2013).

Interestingly, in our study, we observed that some ADRs occur in a cross-sectional manner in patients on antiretroviral therapy such as gastrointestinal upset and headache, and other ADRs are specific for a determinate group of patients; it is very relevant that certain ADRs are significantly associated with a specific therapy such as CNS ADR-EFV and hyperbilirubinemia-ATV. As statistically it is demonstrated that CNS ADRs are practically specific in patients treated with EFV, pharmacogenetic analysis was performed, and it was demonstrated that the presence of *CYP2B6* c.516G>T (rs3745274) is significantly associated with the development of toxicity at the CNS level. The same situation was observed with ATV where we demonstrated a statistical significant association between moderate and severe hyperbilirubinemia (total bilirubin level >1.9 mg/dl) and UGT1A1*28. Conversely, despite gastrointestinal ADRs being common within antiretroviral therapies (Bernal Ortiz et al., 2013), when we performed univariate logistic regression analysis in the dominant, codominant, and recessive model for *CYP2B6* c.516G>T (rs3745274) and UGT1A1*28, we were not able to visualize a statistically significant relationship. Similarly, no association was observed among headache and fever, and a given genotype of variants studied.

On the other hand, adverse reactions at the CNS level, analyzed as a whole or separately, as in the case of nightmares, are widely documented, although there is controversy regarding their appearance (10–74%) and the discontinuation of therapies (2–11%) due to these events (Kenedi and Goforth, 2011; Rosenblatt et al., 2017). In the univariate model of codominant inheritance for the nightmare event, the association with T/T genotype of *CYP2B6* c.516G>T (rs3745274) was observed with efavirenz (OR = 12, *p* = 0.031). Similarly, when we performed the univariate analysis for the recessive inheritance model, an association with the T/T genotype was observed (OR = 7.14, *p* = 0.042) but not with the dominant inheritance model (OR = 3.6, *p* = 0.142), possibly attributable to the low number of recruited patients.

Despite the fact that some ADRs such as insomnia, anxiety, and suicide attempt failed to have a statistically significant association in the univariate statistical models, when grouping them, including nightmares, as CNS ADRs, associations with the *CYP2B6* c.516G>T (rs3745274) T/T genotype and the toxicity, both in the codominant and recessive models, were observed (OR = 30.00, *p* = 0.011; OR = 14.99, *p* = 0.021, respectively), but not in the dominant model (OR = 5.08, *p* = 0.061).

In the present research, we observe a frequency of 0.36 for UGT1A1*28, which is comparable to the 0.33 reported in a previous study on the prevalence of Gilbert's syndrome in the Chilean population, and where the presence of the UGT1A1*28 allele was determined indirectly by analyzing the rs6742078 (G > T) variant located in intron 1 of the *UGT1A1* gene, which is also in strong linkage disequilibrium with UGT1A1*28 (Méndez et al., 2013). These frequencies are very similar to those described in other Latin American (Colombia: 0.34; Peru: 0.45; Mexico: 0.37) and European (0.30; Project 1000 genomes) populations, but different from Asian populations, where allele frequencies of 0.16 have been reported (Atasilp et al., 2020). The genotype frequencies observed in this study for *CYP2B6* c.516G>T (rs3745274) (G/G: 38.81%; G/T: 46.27%; T/T: 14.92%, Table 3) were very similar to those of a previous study carried out in a Chilean population under therapy with EFV (G/G: 43%; G/T: 42%; T/T: 15%) (Carr et al., 2010), where the frequency of allele T (0.38) was very similar to that of other Latin American populations such as Colombian (0.37), Mexicans (0.31), and Puerto Rican (0.35), and higher than that described in Europeans (0.24; Project 1000 genomes). Given the frequencies observed for *CYP2B6* c.516G>T and UGT1A1*28 in Chile and Latin America, it is necessary to consider promoting studies that allow validating the recommendations of the CPIC guidelines for ATV (Gammal et al., 2016) and EFV (Desta et al., 2019) in our countries. These recommendations not only suggest the use of other alternative drugs, to avoid toxicity reactions in patients with genotypes associated with poor metabolizer phenotypes (e.g., homozygous genotype for *CYP2B6* c.516G>T), but also suggest lower initial doses of the same drug (e.g., starting efavirenz with a reduced dose of 400 or 200 mg/day; Desta et al., 2019).

Although this study reached a small sample size, statistically significant associations were observed between specific RAMs for ATV and EFV, with the variants *CYP2B6* c.516G>T and UGT1A1*28, respectively, demonstrating that these studies are necessary: a need that has been highlighted in previous national studies where the association of genetic variants in *CYP2B6* with elevated plasma levels of EFV was evidenced (Carr et al., 2010).

The present study has some shortcomings. The relatively small sample size of examined patients could mask potential associations. Moreover, some other potentially candidate genes/polymorphisms with a low level of evidence (e.g., *CYP3A4/5*, *CYP2A6*, and *SLCO1B1*) were not evaluated, but could be still relevant. Besides, some missing clinical values and possible differential misclassification bias could be relevant, affecting estimated associations between potentially relevant combinations of risk factors and adverse reactions.

To our knowledge, this is the first Latin American study performed to evaluate association between these genetic variants and ADRs in antiretroviral therapy, which allows us to initially characterize the therapeutic behavior in Chilean patients living with HIV.

## Conclusion

Our findings suggest that after treatment with ATV or EFV, UGT1A1*28 and *CYP2B6* c.516G>T (rs3745274) genetic polymorphisms influence the appearance of moderate-to-severe hyperbilirubinemia and CNS toxicity, respectively. Larger prospective studies will be necessary to validate these associations in our population; however, this preliminary study validates the recommendations of the CPIC guidelines to use the pharmacogenetic criteria in prescription of EFV or ATV to patients.

Without a doubt, improving adherence in patients living with HIV is a critical issue for the success of therapy. We must also rescue and optimize the use of drugs classified as “unsafe” whose prescription has decreased, given the type and frequency of adverse reactions they can generate. Many times, their application is strongly questioned despite being very effective. However, pharmacogenetics studies show that the generation of ADRs is not only an effect caused by the drug but rather the relationship with the patient. In this way, clinically, high effective drugs could be kept in force by following the recommendations of pharmacogenetic clinical guidelines by, for example, reducing the prescribed standard doses according to patients’ genotype.

Hence, validating and applying international pharmacogenetics recommendations in Latin American countries would improve the precision of ART: a fundamental aspect to achieve the 90–90–90 treatment target proposed by UNAIDS.

## Data Availability

The original contributions presented in the study can be requested from the corresponding author.
